# Evaluation of Measurement Errors in Rotational Stitching, One-Shot, and Slot-Scanning Full-Length Radiography

**DOI:** 10.3390/bioengineering12090999

**Published:** 2025-09-19

**Authors:** Zhengliang Li, Jie Xia, Cong Wang, Zhemin Zhu, Fan Zhang, Tsung-Yuan Tsai, Zhenhong Zhu, Kai Yang

**Affiliations:** 1 School of Biomedical Engineering and Med-X Research Institute, Shanghai Jiao Tong University, Shanghai 200030, China; lizhengliang@sjtu.edu.cn; 2 Engineering Research Center of Digital Medicine and Clinical Translation, Ministry of Education, Shanghai 200030, China; 3 Department of Radiology, Shanghai Sixth People’s Hospital Affiliated to Shanghai Jiao Tong University School of Medicine, No. 600 Yi Shan Rd., Shanghai 200233, China; 1710208xj@buaa.edu.cn; 4 TAOiMAGE Medical Technologies Corporation, Shanghai 200120, China; wangcongbme@163.com (C.W.); 5140829016@alumni.sjtu.edu.cn (Z.Z.); zhangfan@taoimage.com (F.Z.); 5 Department of Orthopaedics, Shanghai Sixth People’s Hospital Affiliated to Shanghai Jiao Tong University School of Medicine, No. 600 Yi Shan Rd., Shanghai 200233, China

**Keywords:** full-length radiography, slot-scanning, rotational stitching, one-shot imaging, measurement accuracy, quantitative assessment

## Abstract

Full-length radiography is essential for evaluating spinal deformities, limb length discrepancies, and preoperative planning in orthopedics, yet the measurement accuracy of different radiographic methods remains unclear. This phantom study compared the accuracy of rotational stitching, one-shot and slot-scanning full-length radiography across six radiographic systems in quantifying distances between anatomical landmarks. Measurement errors were statistically analyzed using appropriate nonparametric tests. The results demonstrated significant differences in measurement accuracy among the three methods (H (2) = 15.86, *p* < 0.001). Slot-scanning exhibited the highest accuracy, with a mean error of −1.19 ± 10.13 mm, while both rotational stitching and one-shot imaging showed greater systematic underestimation, with mean errors of −18.95 ± 13.77 mm and −15.32 ± 12.38 mm, respectively. These negative biases (approximately 1.9 cm and 1.5 cm) are clinically meaningful because, if unrecognized, they can alter mechanical axis estimation and alignment planning in procedures such as high tibial osteotomy (HTO). Post hoc analysis confirmed the superior accuracy of slot-scanning compared to the other two methods, while no significant difference was found between rotational stitching and one-shot imaging. These findings indicate that system choice substantially impacts measurement accuracy, supporting preferential use of slot-scanning when precise quantitative assessment is required.

## 1. Introduction

Full-length standing radiography is routinely employed to (i) characterize global and regional spinal deformity [1], (ii) quantify leg length discrepancy (LLD), and (iii) support preoperative planning of HTO [2]. To translate these indications into measurable endpoints, standardized alignment parameters are applied. Key spinal metrics include the coronal offset between the C7 plumb line and the central sacral vertical line (CSVL), where an offset >20 mm indicates coronal imbalance [3], and the sagittal vertical axis (SVA), with ≤40 mm generally considered normal or mild and >40 mm denoting clinically relevant sagittal malalignment in adult classification systems [4,5]. For the lower extremities, commonly cited LLD thresholds (<5 mm negligible; ≥5 mm documentable; ≥10 mm functionally consequential; ≥20 mm often prompting orthotic or surgical consideration) guide interpretation [6,7,8]. In HTO planning, varus knees often present a medial mechanical axis deviation (MAD) of approximately 10–30 mm preoperatively [9,10]. Measurement error approaching these categorical boundaries can reclassify deformity severity or alter correction targets, underscoring that clinical utility depends on high geometric fidelity across the entire cranio-caudal field—not merely visually acceptable stitched continuity. Against this methodological backdrop, the primary technologies for full-length radiography—digital stitching [11], slot-scanning [12], and one-shot imaging [13]—differ in acquisition geometry, workflow, cost, and inherent error mechanisms. These differences motivate a comparative evaluation of their measurement accuracy.

Digital stitching techniques for full-length radiography can be broadly classified into three categories: linear stitching, wide stitching, and rotational stitching [14]. Linear stitching involves the simultaneous linear movement of both the X-ray source and the imaging detector along the cranio-caudal axis of the subject, with their relative positions remaining constant throughout the scan. In rotational stitching, the detector moves linearly along the subject, while the center of the X-ray source remains fixed and rotates in synchronization with the detector’s position. Wide stitching is characterized by the linear movement of the detector along the cranio-caudal axis, with the spatial position of the X-ray source kept stationary. All these methods reconstruct a full-length image by aligning and stitching together the overlapping regions obtained from multiple exposures [15]. However, because they rely on sequential acquisitions, they are susceptible to parallax-induced local scaling differences, inter-segment misregistration, and motion between exposures, potentially introducing subtle longitudinal underestimation or localized duplication/omission [16,17].

Rotational stitching offers significant advantages over linear stitching. Although linear stitching systems are relatively easy to develop, the reconstructed images may exhibit duplication or omission of anatomical structures, resulting in inferior image quality compared to rotational stitching. In addition, linear stitching requires the acquisition of a large number of images, leading to a minimum examination time of 21 s, which is longer than that required for rotational stitching (<20 s) [14]. This prolonged duration increases the risk of stitching errors caused by patient movement. Rotational stitching requires coordinated rotational and linear tube movements; recent advances have reduced this engineering barrier and facilitated widespread adoption. This more elaborate trajectory, however, places greater emphasis on maintaining geometric calibration so that minor parallax-related scaling differences or segment-to-segment registration drift do not accumulate over extended cranio-caudal distances [17]. Its versatility—including motorized oblique projections—further contributes to clinical prevalence, and it has become the most commonly used method for full-length radiography [18]. Therefore, rotational stitching equipment was included in this study.

One-shot imaging represents an advancement over wide stitching [19]. The necessity for detector movement in wide stitching is primarily a compromise due to limitations in detector size. With recent progress in digital detector manufacturing, large-area detectors have become feasible, giving rise to the one-shot technique. In this approach, the X-ray source remains stationary, and a single large detector (e.g., 120 × 43 cm) replaces the need for a smaller, movable detector, enabling full-length imaging with a single exposure. This method effectively eliminates stitching errors and motion artifacts. However, due to the nature of cone-beam X-ray projection, only objects located on the central ray path are imaged with true-to-size accuracy [20]. As the distance from the center increases, geometric magnification and distortion become more pronounced, resulting in significant edge distortion with large-area detectors and posing challenges for subsequent image calibration [21]. These peripheral effects can generate non-uniform scaling of inter-landmark distances and, if incompletely corrected, contribute to systematic measurement bias.

Slot-scanning technology was developed to minimize geometric distortion by narrowing lateral divergence. In slot-scanning, the X-ray source generates a very narrow beam (the “slot”), which irradiates only a small portion of the subject at any given time. The slot-shaped X-ray beam and the detector move synchronously along the longitudinal axis of the subject, scanning from one end to the other. Each exposure captures only a small segment of the image, and all segments are sequentially stitched together to form a complete, full-length image. Because the exposed region at any given time is extremely narrow and deviates minimally from the center of the imaging field, slot-scanning can produce full-length images with negligible geometric distortion [1]. Residual variation may still originate from tube–detector synchronization tolerances, cumulative mechanical drift, or patient motion during longer scans; however, these influences are theoretically limited compared with peripheral distortion in cone-beam imaging or parallax introduced by multi-segment stitching.

Given the respective technical advancements and clinical relevance of these methods, this study focuses on three techniques: rotational stitching, one-shot imaging, and slot-scanning. Rotational stitching offers a balance of image quality, acquisition time, and versatility (e.g., oblique capability), yet—because it remains a stitching paradigm—it still inherits multi-exposure geometry sensitivities, including parallax-induced local scaling shifts and motion/inter-segment registration drift that must be tightly calibrated to prevent cumulative bias. One-shot imaging eliminates stitching, thereby avoiding multi-exposure motion and registration accumulation, yet leaves unresolved peripheral cone-beam magnification and nonuniform scaling that can yield edge-region systematic deviation if incompletely corrected. Slot-scanning was engineered to suppress geometric distortion by maintaining each strip near the effective beam center; its residual error profile instead shifts toward synchronization tolerance and residual synchronization drift (tube–detector timing, mechanical translation stability) plus potential prolonged-scan motion. Nevertheless, the comparative magnitude and direction of these systematic and random components under standardized geometric conditions remain insufficiently quantified, constraining evidence-based modality selection where sub-centimeter accuracy is required.

Although full-length radiography is rapidly developing and widely used, the extent and sources of measurement errors for each method remain unclear, which can have significant clinical consequences. Inaccurate imaging may lead to misclassification of spinal deformities [22], flawed assessment of limb discrepancies [23], or compromised surgical planning—potentially resulting in suboptimal treatment and outcomes. Therefore, systematically quantifying the accuracy and error sources of full-length imaging systems under standardized conditions is essential. Such evidence-based evaluations can help clinicians choose the most appropriate modality for specific needs and support manufacturers in improving imaging technologies and calibration methods.

In this study, we aim to address this gap by conducting a comprehensive analysis of six full-length radiographic systems. Specifically, we focus on evaluating their geometric distortion and measurement stability. By quantifying the accuracy and reliability of these imaging systems, this research not only supports clinicians in making informed decisions but also contributes to advancing technological innovation and promoting standardization in clinical imaging practices. Ultimately, this study seeks to enhance diagnostic accuracy, improve surgical outcomes, and ensure the consistency and reliability of imaging-based measurements in clinical applications.

## 2. Materials and Methods

### 2.1. Phantom Preparation and Reference Standard Establishment

**Figure 1:**
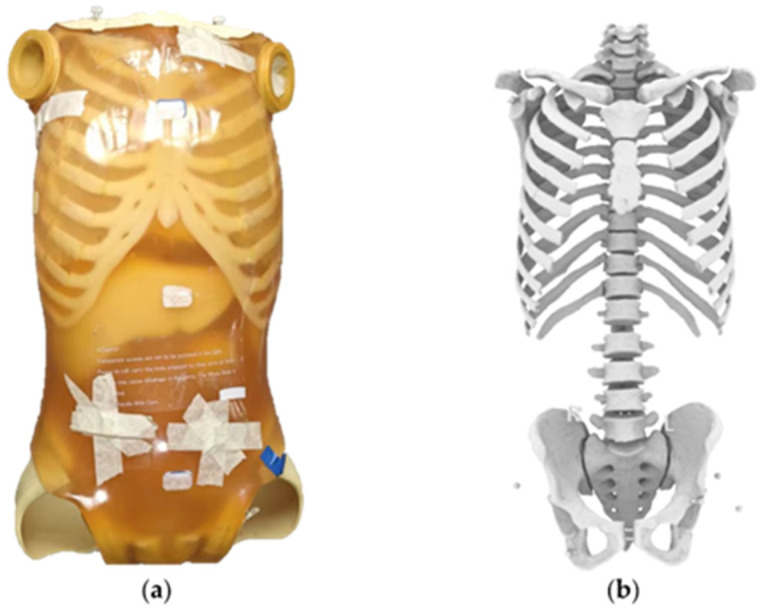
(**a**) Whole-body phantom with steel beads affixed at anatomical landmarks; (**b**) Corresponding skeletal structure and marker segment from the CT volume.

A whole-body anthropomorphic phantom specifically designed for conventional X-ray projection training and geometric evaluation (PBU-50, Kyoto Kagaku Co., Ltd., Kyoto, Japan; Figure 1a), with radiological attenuation and CT Hounsfield values approximating those of the human body, was employed. Extensive prior studies have documented the broad application of this series of phantoms in CT dose optimization, shielding efficacy assessment, radiographic positioning accuracy analyses, and protocol training [24,25,26]. The phantom is composed of a urethane-based soft tissue equivalent (density 1.06 g/cm^3^), synthetic bone regions molded from epoxy resin (density 1.31 g/cm^3^) approximating a composite cortical–cancellous response, and a lower-density epoxy skull component (density 1.11 g/cm^3^). Sufficient radiographic differentiation for reliable steel bead centroid localization without excessive beam hardening is afforded by these material contrasts. For radiographic acquisitions, the phantom was firmly secured on a custom plain wooden support frame to ensure reproducible positioning while avoiding metal-induced artifacts. Steel beads (8.00 mm diameter) were affixed at key anatomical landmarks using a minimal amount of neutral cyanoacrylate adhesive to ensure positional stability while avoiding perceptible additional attenuation, so that subsequent geometric measurements could be performed. After marker placement, the phantom was scanned on a uCT 960+ CT system (United Imaging Healthcare, Shanghai, China) using 120 kV, 1 mm slice thickness, and 0.80 × 0.80 mm in-plane pixel spacing; the reconstructed images (Figure 1b) were used to extract three-dimensional bead coordinates, which were taken as the gold-standard reference for all ensuing measurement accuracy analyses.

Six different radiographic systems, representing three radiographic methods—rotational stitching, one-shot and slot-scanning—were used to obtain whole-body X-ray images of the phantom. The accuracy of each system was evaluated by comparing the distances measured between anatomical markers on the two-dimensional full-length X-ray images with those obtained from the CT-based gold standard. The radiographic systems and their key technical parameters are summarized in Table 1.

### 2.2. Fix Markers

**Figure 2:**
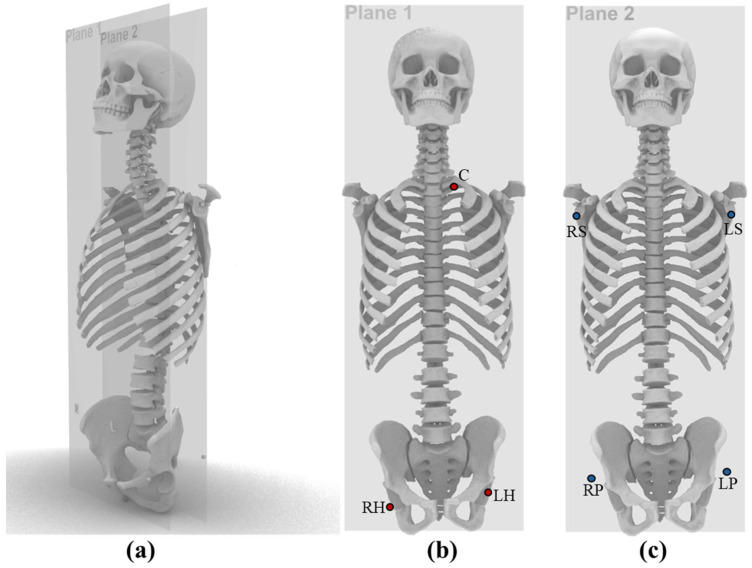
(**a**) Schematic showing two coronal landmark planes (Plane 1 and Plane 2) positioned at different depths within the anthropomorphic phantom; the planes are separated by approximately 7.5 cm along the longitudinal (cranio-caudal) axis; (**b**) Landmarks on Plane 1 (red dots); (**c**) Landmarks on Plane 2 (blue dots).

To avoid errors caused by anatomical landmark localization, steel beads with a diameter of 8.00 mm were fixed at seven locations on the phantom, including the chest (C), left shoulder (LS), right shoulder (RS), left pelvis (LP), right pelvis (RP), left hip (LH), and right hip (RH). To minimize projection errors as much as possible, the C, RH, and LH beads were positioned approximately within plane 1, while the RP, LP, LS, and RS beads were positioned approximately within plane 2. Plane1 and plane2 are coronal planes at different depths. (Figure 2).

In this study, the distances between the centers of the steel beads within each plane were measured, resulting in a total of 9 unique marker pair distances, to simulate clinical measurements of anatomical reference distances.

### 2.3. Three-Dimensional Measurement of Anatomical Landmark Distances Using CT

A MATLAB 2024 program was developed to import the CT volume of the phantom. Using threshold segmentation, the bones and steel beads were extracted (Figure 1b). The centers of all steel beads were fitted as spheres, and the fitted centers were used as the spatial coordinates of anatomical landmarks in the CT coordinate system. The distances between these anatomical landmarks were then measured.

### 2.4. Two-Dimensional Measurement of Anatomical Landmark Distances on Full-Length Radiographs

**Figure 3:**
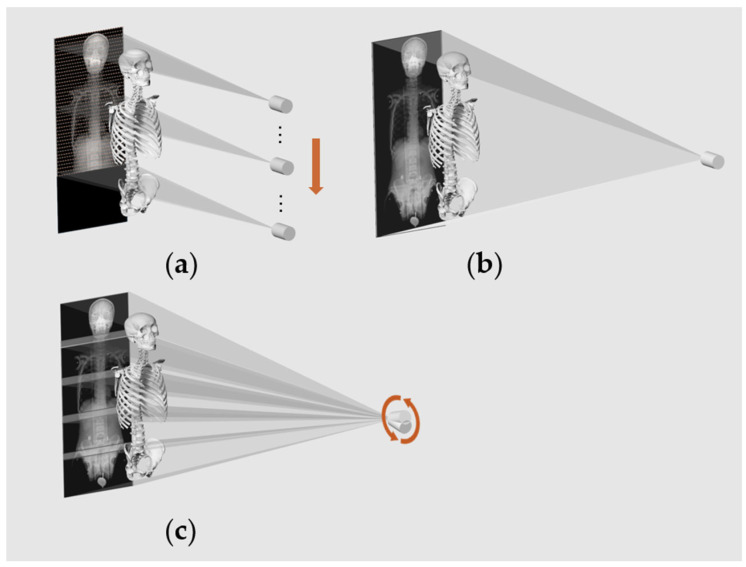
Three methods used for full-length radiography (**a**) Slot-scanning: The X-ray source and detector move vertically together. A narrow X-ray beam scans the object, creating a full-length image.; (**b**) One-shot imaging: The X-ray source captures the entire phantom in a single exposure on a 1200 mm × 600 mm flat panel detector.; (**c**) Rotational stitching: The X-ray source rotates while the detector moves downward, acquiring multiple overlapping images, which are subsequently stitched together based on the overlapping regions. The arrows indicate the direction of movement of the X-ray source.

We obtained full-length images using three methods: rotational stitching (UDR780IPRO3F, GC85A, DRX-Compass) and one-shot imaging, slot imaging (EOS imaging, TAOiMAGE) (Figure 3). During the acquisition of full-length images, the spatial position of the static phantom and the imaging system remained unchanged. The images from multiple exposures were almost identical, so only one full-length image was used to represent each imaging device in this study. During exposure, the coronal plane of the phantom was kept parallel to the imaging plate.

### 2.5. Fitting the Centers of Steel Beads

**Figure 4:**
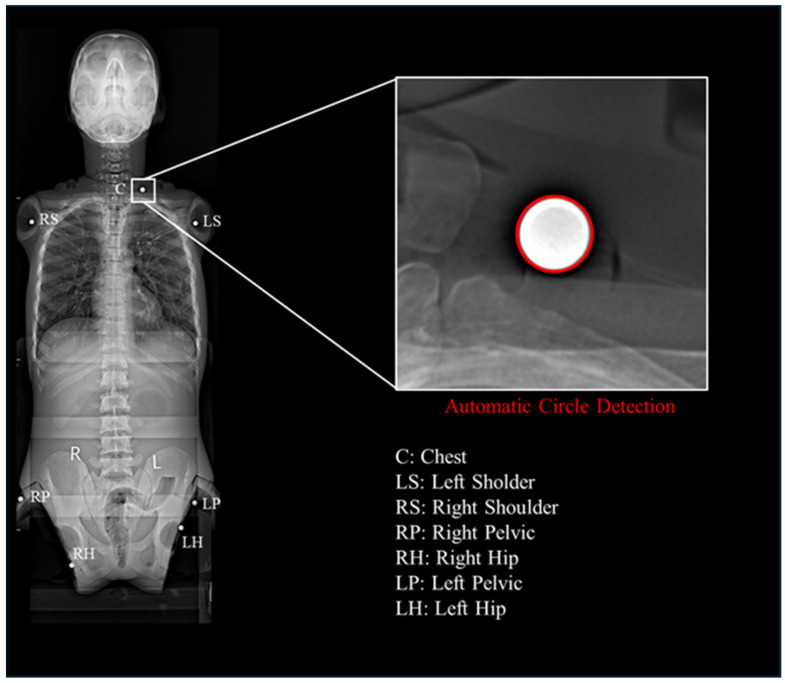
Automatic fit the centers of the steel beads on full-length x ray image.

A MATLAB interactive interface was developed to fit the centers of the steel beads. After importing and displaying the full-length X-ray image, regions containing steel beads were manually selected to reduce background interference. Within each selected region, Canny edge detection was applied, and the edge points were used in the Hough transform to detect circles (Figure 4). The pixel diameter $R_{\mathrm{pixel}}$, center coordinates and a circularity metric were recorded for each detected circle. The metric is defined as the number of votes for a circle divided by the total number of votes in the accumulator. A higher metric indicates stronger support for the presence of a circle.

### 2.6. Scaling Correction

This study investigates systematic errors introduced by different full-length radiographic techniques and their correction methods. To compensate for scaling error due to source-to-image distance (SID) and source-to-object distance (SOD), scaling correction using steel beads was performed. The actual diameter of each steel bead was measured with a caliper ($R^{*}$ = 8.00 mm).

On each radiograph, the steel bead diameter was calculated as:

(1) $$R_{\mathrm{image}\;}=R_{\mathrm{pixel}}\times \mathrm{spacing}$$

where $R_{\mathrm{pixel}}$ is the measured diameter in pixels, and “spacing” denotes the pixel size (mm/pixel).

The C, LH, and RH are closer to the X-ray source, resulting in a greater magnification and thus larger bead diameters, whereas RP, LP, LS, and RS are closer to the imaging plate, leading to a smaller magnification. Therefore, in this study, the seven points were divided into two planes, and the mean value of the bead diameters within each plane was calculated as the calibration factor to correct the distances within that plane. Calibration coefficients $\varepsilon_{i}$(i = 1, 2) for each plane were then calculated as follows:

(2) $$\varepsilon_{i}=\frac{R_{plane\_i}^{*}}{R^{*}}$$

where $R_{plane\_i}^{*}$ represents the mean measured diameter of all steel beads in plane_i (i = 1, 2).

For distance measurements, the Euclidean distance between markers on the image ($l_{\mathrm{pixel}}$, in pixels) was first determined. The actual distance between a pair of markers, l, was then calculated as follows:

(3) $$l=\frac{l_{\mathrm{pixel}}\times \mathrm{spacing}}{\varepsilon_{i}}$$

Notably, all measurements were based on images that underwent dual calibration: geometric distortion was first corrected using each manufacturer’s proprietary image protocol, followed by the application of a steel bead calibration method to eliminate magnification effects caused by variations in the phantom-to-detector distance. Therefore, the measurement errors observed in this study (final error = original image error × image protocol correction × steel bead correction) reflect the highest level of accuracy attainable under our experimental conditions. Given that the steel bead calibration method was applied uniformly across all radiographic systems, the observed inter-system differences in measurement accuracy can be primarily attributed to two key factors: the intrinsic error characteristics of the original images produced by different radiographic methods, and the effectiveness of each manufacturer’s image protocol correction algorithm.

### 2.7. Accuracy Comparison

To compare the accuracy of different full-length imaging devices, the distances measured on CT images were selected as the gold standard. First, the voxels containing the steel beads were segmented using a thresholding method. The centers of these voxels were then fitted to obtain the coordinates of the sphere centers. The distance between two points, defined as the connection between the centers, was used as the gold standard ($l_{\mathrm{CT}}$). The measurement error for each device was calculated as follows:

(4) $$Error=l_{\mathrm{full}\mathrm{length}}-l_{\mathrm{CT}}$$

In this study, the MAE (Mean Absolute Error), RMSE (Root Mean Square Error), SD (Standard Deviation), and Bias of the Error were calculated. These statistical metrics were chosen to comprehensively evaluate the characteristics of measurement errors for each device. MAE reflects the average magnitude of deviation between the measured results and the gold standard, with smaller values indicating higher overall accuracy. RMSE places greater emphasis on larger errors, thus highlighting the impact of outliers on overall accuracy. SD quantifies the dispersion of the errors; a smaller SD indicates more consistent measurement results. Bias represents the average direction of the error: a positive bias suggests that the measurements tend to overestimate, while a negative bias indicates a tendency to underestimate; a bias close to zero implies no systematic deviation. By collectively analyzing these four statistical metrics, the measurement accuracy and consistency of different devices can be compared more objectively and comprehensively.

### 2.8. Statistical Methods

All statistical analyses were performed using MATLAB 2024. Measurement errors for each radiographic system were first assessed for normality using the Shapiro–Wilk test. For group comparisons, Levene’s test was applied to evaluate the homogeneity of variances.

To determine whether the measurement errors of each radiographic system significantly deviated from zero, we selected statistical tests based on the distribution characteristics. For systems with normally distributed errors (Shapiro–Wilk *p* > 0.05), both one-sample *t*-tests and Wilcoxon signed-rank tests were used to assess whether the mean error differed significantly from zero. For systems not meeting the normality assumption (Shapiro–Wilk *p* ≤ 0.05), only the non-parametric Wilcoxon signed-rank test was applied. Statistical significance was defined as *p* < 0.05.

To investigate the influence of imaging methods on measurement precision, the six radiographic systems were categorized into three groups according to their imaging principles: rotational stitching, one-shot and slot-scanning. The normality of measurement errors within each group was again assessed using the Shapiro–Wilk test, and Levene’s test was used to verify homogeneity of variances across groups. If the normality assumption is not met, group differences were analyzed using the non-parametric Kruskal–Wallis test. Post hoc pairwise comparisons were performed using Dunn’s test with Sidak adjustment

Descriptive statistics, including mean, standard deviation (SD), and median, were calculated for each group. Boxplots were generated to visualize the distribution and variability of measurement errors among different radiographic systems and methods.

All statistical tests were two-sided, and statistical significance was set at *p* < 0.05.

## 3. Results

### 3.1. Steel Beads Diameter Measurements

Across imaging systems, the plane-averaged steel bead diameters deviated systematically from the nominal 8.00 mm (Table 2). In Plane 1, means ranged from 7.96 mm (EOS; bias −0.04 mm, −0.5%) to 11.90 mm (DRX-Compass; +3.90 mm, +48.8%), with intermediate positive biases for DSI-DRP (+0.69 mm, +8.6%), GC85A (+1.25 mm, +15.6%), Eagle Eye (+1.69 mm, +21.1%), and UDR780iPro3F (+2.04 mm, +25.5%). Plane 2 showed the same ordering (EOS: 7.86 mm, −1.8%; DRX-Compass: 11.53 mm, +44.1%), and the other systems exhibited +6.9% to +19.8% enlargement. Notably, every system measured Plane 1 beads larger than Plane 2, indicating a consistent depth-related magnification effect. Inter-plane consistency, expressed as $∆\varepsilon =\varepsilon_{P1}-\varepsilon_{P2}$, was high for Eagle Eye (∆ε = 0.01) and EOS (∆ε = 0.02), but lower for GC85A (∆ε = 0.09), indicating greater sensitivity to geometric setup.

The diameter of the steel bead was calculated as follows:

**Table 2:** Measured Diameters of Steel Beads Across Radiographic Systems.

Markers		Diameter of Steel Beads Measured by Different Systems(mm)					
		Eagle Eye	EOS	GC85A	UDR780iPro3F	DRX-Compass	DSI-DRP
Plane 1	C	9.69	7.92	9.14	9.93	11.92	8.67
	LH	9.64	7.96	9.30	10.04	11.84	8.76
	RH	9.75	8.01	9.30	10.13	11.94	8.65
	MEAN	9.69	7.96	9.25	10.04	11.90	8.69
	ε	1.21	1.00	1.16	1.25	1.49	1.09
Plane 2	RP	9.59	7.88	8.74	9.72	11.75	8.57
	LP	9.64	7.88	8.70	9.51	11.79	8.53
	LS	9.58	7.85	8.47	9.45	11.29	8.50
	RS	9.53	7.82	8.44	9.41	11.27	8.60
	MEAN	9.58	7.86	8.58	9.52	11.53	8.55
	ε	1.20	0.98	1.07	1.19	1.44	1.07

### 3.2. Hough Transform Circularity Metric Results

The Hough transform circularity metrics for steel beads ranges from 0 to 1, with values closer to 1 indicating higher image circularity and reduced distortion (Table 3).

Mean circularity metrics ranged from 0.36 to 0.61. EOS Imaging achieved the highest mean value (0.61 ± 0.08), followed by DSI-DRP (0.60 ± 0.05) and Eagle Eye (0.60 ± 0.05). DRX-Compass had the lowest mean (0.36 ± 0.11), indicating greater image distortion.

Regarding measurement stability, UDR780IPRO3F demonstrated the highest spatial consistency (SD = 0.04), followed by Eagle Eye (SD = 0.05) and DSI-DRP (SD = 0.05). DRX-Compass showed both the lowest mean and the greatest variability (SD = 0.11), reflecting substantial differences in image quality across spatial positions.

### 3.3. Distance Error of Different Manufacturers

There are notable differences in measurement accuracy and consistency among the various manufacturers (Table 4). Eagle Eye and EOS Imaging demonstrated the lowest measurement errors, outperforming the other systems. Notably, Eagle Eye achieved the best results in root mean square error (RMSE, 9.31 mm), mean absolute error (MAE, 8.05 mm), and standard deviation (SD, 9.09 mm), indicating superior overall accuracy and consistency. In contrast, EOS Imaging exhibited the lowest bias (−0.34 mm), reflecting minimal systematic deviation from the true value. EOS also showed favorable RMSE (10.49 mm), MAE (9.30 mm), and SD (10.48 mm), although these were slightly higher than those of Eagle Eye.

DroidSurg Medical DSI-DRP ranked intermediately among the evaluated systems, with an RMSE of 19.26 mm, MAE of 15.32 mm, bias of −15.32 mm, and SD of 11.67 mm. These metrics indicate higher accuracy and consistency compared to UIHUDR780IPRO3F, Samsung HealthcareGC85A, and Carestream Health DRX-Compass, though it remained less precise than TAOiMAGE Eagle Eye and EOS Imaging. While DroidSurg Medical DSI-DRP showed a systematic underestimation, its error magnitude and variability were lower than those of UIHUDR780IPRO3F (bias: −20.13 mm), Samsung HealthcareGC85A (bias: −19.78 mm), and Carestream Health DRX-Compass (bias: −16.93 mm). Among these, DRX-Compass exhibited the highest variability (SD: 16.24 mm), indicating reduced reliability relative to the CT reference standard.

In summary, Eagle Eye and EOS Imaging demonstrated superior accuracy and consistency in distance measurement, closely aligning with the CT gold standard and exhibiting minimal systematic and random errors. DSI-DRP showed intermediate performance, with measurement characteristics falling between the leading and lower-performing systems. UDR780IPRO3F, GC85A, and DRX-Compass were characterized by more substantial systematic underestimation and greater error variability.

### 3.4. Statistical Analysis

#### 3.4.1. Significance of Measurement Errors Across Radiographic Systems

Normality of the error distributions for each radiographic system was assessed using the Shapiro–Wilk test (Table 5). The results indicated that all systems except DSI-DRP exhibited normal distributions (*p* > 0.05).

For Eagle Eye and EOS imaging, neither the *t*-test nor the Wilcoxon test indicated significant deviation (all *p* > 0.05), suggesting no systematic bias in these systems (Eagle Eye: *t*-test *p* = 0.545, Wilcoxon *p* = 0.570; EOS imaging: *t*-test *p* = 0.929, Wilcoxon *p* = 0.910). In contrast, UDR780IPRO3F, GC85A, and DRX-Compass showed significant deviations from zero in both tests (all *p* < 0.05), indicating the presence of systematic errors. For DSI-DRP, which did not meet the normality assumption, only the Wilcoxon signed-rank test was applied. The results revealed a significant deviation from zero (Wilcoxon *p* = 0.004), suggesting a systematic bias in this system as well.

In summary, Eagle Eye and EOS imaging demonstrated no significant systematic error, while UDR780IPRO3F, GC85A, DRX-Compass, and DSI-DRP exhibited significant systematic deviations in measurement error.

**Figure 5:**
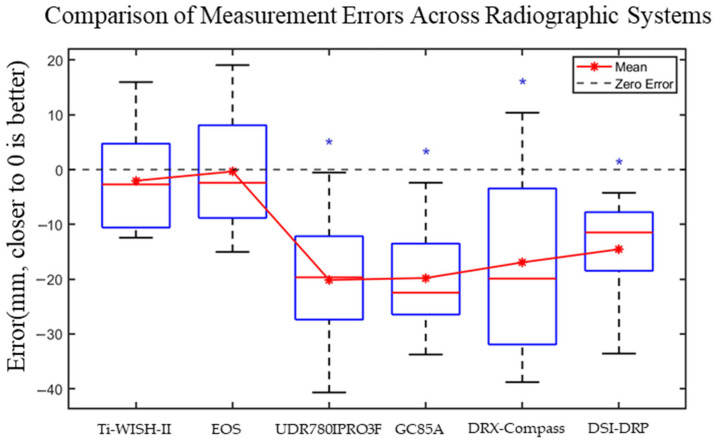
Error distribution across radiographic systems. Boxplots show registration errors (mm); red stars indicate mean values. Blue asterisks denote significant deviation from zero (*p* < 0.05, Wilcoxon signed-rank test). Each system’s boxplot is based on the same sample size (*n* = 9).

Boxplot analysis revealed substantial variation in measurement error among the radiographic systems (Figure 5)**.** Both Eagle Eye and EOS Imaging exhibited minimal measurement bias, with mean errors closely approximating zero and no statistically significant deviation from the zero-error reference. In contrast, UDR780IPRO3F, DRX-Compass, GC85A, and DSI-DRP consistently underestimated distances, as indicated by mean errors significantly below zero (*p* < 0.05, Wilcoxon signed-rank test).

#### 3.4.2. Influence of Imaging Methods on Measurement Error

Measurement error distributions were summarized using medians and interquartile ranges (IQR): Slot-scanning median = −2.56 mm (IQR = [−9.63, 6.08]), Rotational Stitching median = −19.89 mm (IQR = [−29.98, −3.98]), and One-Shot median = −11.87 mm (IQR = [−19.19, −7.77]). Sample sizes were Slot-scanning (*n* = 18), Rotational Stitching (*n* = 27), and One-Shot (*n* = 9).

Normality of measurement errors was assessed using the Shapiro–Wilk test. The Slot-scanning group (*p* = 0.2374) and the Rotational Stitching group (*p* = 0.2003) both exhibited normal distributions (*p* ≥ 0.05), while the One-Shot group deviated from normality (*p* = 0.0079) Homogeneity of variances was supported (Levene’s test *p* = 0.4988). Because one group was non-normal and group sizes were unequal, a rank-based omnibus test (Kruskal–Wallis) was used. The Kruskal–Wallis test indicated a statistically significant overall difference among systems (H (2) = 15.86, *p* < 0.001). The effect size was $\epsilon^{2}=0.272$, computed as $\epsilon^{2}=(H-k+1)/(N-k)$ with k = 3 groups and total N = 54. This magnitude is considered large based on commonly cited heuristic thresholds for nonparametric omnibus effects.

Post hoc pairwise comparisons were performed using Dunn’s test with Sidak adjustment (m = 3 pairwise contrasts). Adjusted *p*-values were calculated as $p_{adj}=1-{\left(1-p_{raw}\right)}^{m}$, and all *p*-values reported below are Sidak-adjusted. Mean rank differences (MRD) and simultaneous 95% confidence intervals (CI) were: Slot-scanning vs. Rotational Stitching MRD = 18.74, 95% CI [7.31, 30.17], $p_{adj}$ = 0.0003; Slot-scanning vs. One-Shot MRD = 15.44, 95% CI [0.11, 30.78], $p_{adj}$ = 0.0478; Rotational Stitching vs. One-Shot MRD = −3.30, 95% CI [−17.75, 11.16], $p_{adj}$ = 0.9291. For significant contrasts, the entire CI lay above zero; the non-significant contrast spanned zero, consistent with its large adjusted *p*-value. Taken together, the significant omnibus test, large effect size, and the direction of the pairwise confidence intervals indicate that the present sample sizes were adequate to discriminate between systems without yielding unstable or spurious findings.

Descriptive statistics revealed that the slot-scanning method (*n* = 18) achieved the highest measurement accuracy, with a mean error of −1.19  ±  10.13 mm (mean  ±  SD) and a median error of −2.56 mm. Both rotational stitching (*n* = 27) and one-shot methods (*n* = 9) exhibited similar systematic underestimation, with mean errors of −18.95  ±  13.77 mm and −15.32  ±  12.38 mm, and median errors of −19.89 mm and −11.87 mm, respectively.

**Figure 6:**
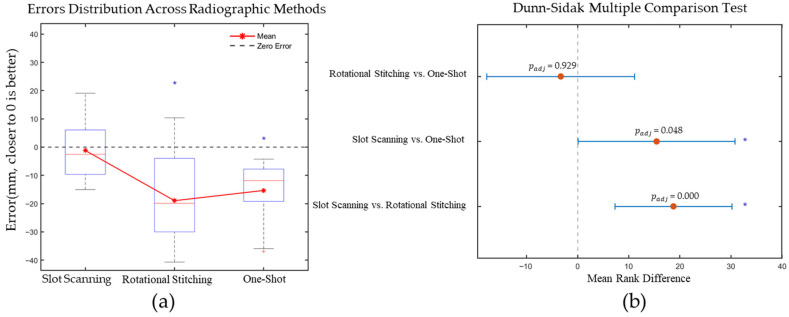
(**a**) Error distribution boxplot of different imaging method. Blue asterisks denote significant deviation from zero (*p* < 0.05, Wilcoxon signed-rank test).; (**b**) Dunn–Sidak multiple comparisons of rotational stitching vs. one-shot imaging, slot-scanning vs. one-shot and slot-scanning vs. rotational stitching. Asterisks (*) indicate statistically significant differences between groups (*p* < 0.05).

As illustrated in the boxplot (Figure 6a), the slot-scanning method demonstrated error distributions that were closer to the zero-error line and exhibited a narrower interquartile range. Rotational stitching showed greater variability, with some measurements approaching zero error. The one-shot method displayed the most concentrated error distribution, but with a consistent negative bias, indicating stable systematic underestimation. The Dunn–Sidak multiple comparison results (Figure 6b) further quantified the statistical differences between groups.

In summary, the underlying imaging principle exerts a significant influence on measurement accuracy. Slot-scanning technology, by acquiring images through continuous scanning, effectively reduces geometric distortion and yields the most accurate measurements. In contrast, rotational stitching and one-shot techniques are more prone to systematic measurement bias due to their inherent imaging geometries.

## 4. Discussion

### 4.1. Measurement Accuracy and Systematic Errors Across Rradiographic Systems

Quantifying the measurement accuracy and error sources of full-length imaging systems is vital for improving clinical decision-making and patient care.

Through a comprehensive evaluation of six radiographic systems under standardized conditions, we identified significant performance differences: TAOiMAGE Eagle Eye and EOS Imaging demonstrated the highest accuracy, with no significant systematic errors (all *p* > 0.05 for *t*-tests and Wilcoxon tests) and mean errors near zero. This near-null bias is concordant with published data showing that slot-scanning EOS systems typically exhibit negligible or slightly negative magnification (≈−0.5% to −0.8%) while conventional CR/DR techniques often present a positive magnification of about 5–8% in lower limb measurements [27,28,29,30].

Conversely, UIHUDR780IPRO3F, Samsung Healthcare GC85A, Carestream Health DRX-Compass, and DroidSurg Medical DSI-DRP displayed significant systematic deviations (all *p* < 0.05), indicating measurement biases that are directionally consistent with the documented overestimation tendencies of non–slot-scanning acquisition geometries [31].

Additionally, the slot-scanning method outperformed rotational stitching and one-shot techniques, achieving the highest accuracy (mean error of −1.19 ± 10.13 mm) and lower geometric distortion (Kruskal–Wallis test, H_2_ = 15.86, *p* < 0.001), corroborating prior reports that slot-scanning biplanar imaging yields improved geometric fidelity and reduces dependence on calibration object positioning compared with traditional projection workflows [32]. These results guide clinicians in selecting reliable imaging systems and modalities while offering manufacturers insights to enhance technologies. They also align our empirical observations with established evidence on magnification control and spatial consistency, while offering manufacturers insights to enhance technologies through mitigation of position-dependent scaling errors [27,30].

### 4.2. Analysis of Inter-Group Errors

#### 4.2.1. Measurement Errors Attributable to Full-Length Radiography Methods

The three full-length radiography methods have different systematic errors due to variations in their imaging principles (Table 6). Among them, rotational stitching and one-shot imaging show clear differences. Rotational stitching uses a movable radiation source and a smaller imaging plate, resulting in a shorter SID, which is practical in the limited space available in hospitals. In contrast, one-shot imaging uses a larger imaging plate, requiring a longer SID (2.1 m). Both methods are based on cone beam projection (Figure 3b,c), and their geometric distortion follows the same pattern: the farther the imaging target is from the center, the greater the distortion; and the larger the SID, the closer the projection is to parallel projection. As a result, one-shot imaging has smaller geometric distortion. Furthermore, one-shot imaging uses a single exposure, which completely avoids stitching errors. From the results, the measurement error of one-shot imaging is significantly lower than that of rotational stitching, which aligns with theoretical predictions.

In contrast, radiographic systems employing slot-scanning technology (such as Eagle Eye and EOS Imaging) achieve image acquisition through the vertical synchronization of a linear detector and a slot collimator, and—critically—avoid the wide cone-beam divergence inherent to the other two modalities. The X-ray source emits a narrow beam through the collimator, which is projected onto the detector as it moves vertically at a constant speed. Throughout the scan, the X-ray beam remains perpendicular to the detector plane, and the geometric relationship among the source, detector, and object is consistently maintained. When contrasted with rotational stitching and one-shot cone-beam full-field acquisition, three domain-relevant advantages emerge:

1. Minimization of Geometric Distortion: The extremely narrow collimation produces a quasi-parallel projection at each incremental position, sharply limiting angular ray divergence. By contrast, the one-shot modality deploys a broad cone beam over a large field of view; peripheral rays impinge at steeper angles, generating systematic anisotropic magnification gradients even with a longer SID. Rotational stitching must register and interpolate overlapping segments acquired under slightly varying projection angles; any minor misregistration or interpolation smoothing can propagate into systematic distance bias.
2. Systematic Reduction in Scattered Radiation: The slot collimator restricts the irradiated area, reducing scattered radiation by over 80% compared to conventional modalities and significantly enhancing image contrast and edge definition [33].
3. Geometric Consistency During Scanning: The fixed geometric relationship and single-axis translation ensure uniform imaging conditions throughout the scanned region. Rotational stitching compounds geometric variability because each partial exposure is acquired at a slightly different source–object–detector geometry along an arcuate or translational path, requiring algorithmic warping and blending that can introduce residual non-linear scaling.

#### 4.2.2. Correction Errors Attributable to Image Protocols and Algorithms

Correction errors arise during post-processing, including system calibration, geometric correction, and image merging. For stitching-based methods, even minor inaccuracies in calibration or alignment can introduce systematic bias, particularly at the image periphery where geometric distortion is most pronounced. These errors may result in local deformation, misalignment, and reduced measurement reliability.

Although one-shot exposure eliminates the need for stitching, the large imaging field exacerbates detector non-uniformity and peripheral distortion. The divergent nature of the X-ray beam and the non-linear detector response at the edges present significant challenges for geometric correction algorithms, limiting their ability to fully compensate for these errors, especially in peripheral regions.

Slot-scanning technology, by avoiding extensive stitching and oversized detectors, simplifies calibration to system synchronization and linear geometric correction. This reduces both the number and magnitude of potential errors, resulting in better accuracy and consistency.

### 4.3. Analysis of Intra-Group Errors

#### 4.3.1. Difference Between EOS Imaging and Eagle Eye

Eagle Eye and EOS demonstrated comparable measurement error, with minor intra-group differences likely attributable to variations in manufacturer control over slit width and edge quality. Notably, the EOS system employs a gaseous X-ray detector (multi-wire proportional chamber) [34], while Eagle Eye utilizes a digital flat-panel dynamic detector; differences in the physical properties of these imaging plates may influence image quality. Before steel bead calibration, the EOS system produced measurements of steel bead diameter that were very close to the true value of 8.00 mm. This high level of accuracy is mainly attributed to the EOS system’s integrated automatic measurement of SID and SOD. Once the technician marks the imaging center, the system can automatically correct for image magnification effects caused by variations in SID or SOD. In contrast, the Eagle Eye system does not have automatic SID and SOD measurement capabilities and therefore cannot perform such corrections automatically, leading to an overestimation of the bead diameter. However, after steel bead calibration, both systems achieved similar measurement accuracy.

#### 4.3.2. Difference Between UDR780IPRO3F, GC85A and DRX-Compass

The MAE, RMSE, bias, and SD for UDR780IPRO3F, GC85A, and DRX-Compass are the same order of magnitude, indicating no significant superiority or inferiority among these radiographic systems.

All manufacturers adopt a workflow in which the region of interest (ROI) is manually set via the software interface, after which the detector and X-ray source move automatically to perform imaging. Therefore, the main sources of error in rotational stitching are stitching errors and mechanical errors arising from the movements of the X-ray source and detector. However, since the stitching algorithms of different manufacturers have not been disclosed, direct comparison is not possible. Mechanical movement errors are also difficult to quantify.

### 4.4. Limitations

This study has several limitations. First, the use of CT 3D distances as the gold standard introduces inevitable projection errors when converting three-dimensional linear distances to two-dimensional projections, despite efforts to fix the steel beads in a plane parallel to the imaging plate. However, this setup mirrors clinical practice, where 2D X-ray images are used to measure anatomical distances with the intent of approximating true 3D spatial distances; thus, the experimental conditions of this study align with real-world clinical scenarios. Second, unlike routine clinical measurements that directly assess distances between anatomical landmarks, we first calibrated 2D image distances using the average diameter of steel beads across two planes. This calibration was performed to isolate measurement error attributable to imaging modality design while minimizing geometric magnification effects, but it may limit direct comparability with studies that do not apply such pre-calibration. Third, the number and spatial distribution of selected steel bead points (corresponding to commonly used landmarks: C, LS, RS, LP, RP, LH, RH) may influence the magnitude and stability of the estimated errors; alternative landmark sets or denser phantoms could yield slightly different error profiles. Finally, because the geometric distortion correction, stitching, and warping workflows are vendor-specific and not fully disclosed, our ability to distinguish the relative contributions of intrinsic acquisition geometry versus downstream algorithmic processing to the residual error is necessarily limited.

Future studies should focus on developing advanced projection correction techniques and standardized calibration protocols to reduce errors, while optimizing landmark selection and integrating these methods into clinical workflows for enhanced precision and practicality. Additionally, evaluating the effectiveness of machine learning or deep learning-based correction methods for compensating image geometric distortions, as well as exploring automated landmark detection algorithms, may further reduce subjective errors associated with manual region selection and improve overall measurement accuracy.

## 5. Conclusions

This study demonstrated that the measurement accuracy of full-length radiography varies significantly among different radiographic methods. Slot-scanning showed the highest accuracy and consistency in quantifying anatomical distances, outperforming both rotational stitching and one-shot imaging, which tended to systematically underestimate measurements. These results suggest that slot-scanning should be preferred in clinical situations where precise quantitative assessment is required. These results suggest that slot-scanning may be advantageous in clinical situations where precise quantitative assessment is required. However, further studies under dynamic conditions and with a broader range of radiographic systems are needed to confirm these findings and guide optimal radiographic system selection in clinical practice.

## Figures and Tables

**Table 1:** Radiographic Systems for Full-length X-ray Imaging Accuracy Comparison.

Systems	Manufactures	Detector Panel Size (mm)	Spatial Resolution (mm)	SID ^2^/SOD ^3^ (mm)	Image Size (pixel)	Imaging Principle	Protocal Name
Eagle Eye	TAOiMAGE, Shanghai, China	430 ^1^	0.139	1230/1500	8268 × 3072	Slot-scanning	FL AP Spine ^4^
EOS	EOS Imaging, Paris, France	340 ^1^	0.179	987/1300	5518 × 1896	Slot-scanning	FL AP Spine ^4^
GC85A	Samsung Healthcare, Suwon, South Korea	430 × 430	0.14	1959/2054	8701 × 2928	Rotational stitching	FL AP Spine ^4^
UDR780iPro3F	United Image Healthcare, Shanghai, China	430 × 430	0.111	1703/1863	10,895 × 3258	Rotational stitching	FL AP Spine ^4^
DRX-Compass	Carestream Health, Rochester, NY, USA	430 × 430	0.139	849/999	9792 × 3010	Rotational stitching	Auto-LLI ^5^
DSI-DRP	DroidSurg Medical, Shanghai, China	430 × 1200	0.139	1900/2100	8704 × 3702	One-shot	SPINE

^1^ Slot length for slot-scanning. ^2^ Source to Image Distance. ^3^ Source to Object Distance. ^4^ Full-length AP View of the Spine. ^5^ Automatic Long Length Imaging.

**Table 3:** Hough Transform Circularity Metrics Across Radiographic Systems.

Markers	Hough Transform Circularity Metric of Different Radiographic System					
	Eagle Eye	EOS Imaging	UDR780iPro3F	GC85A	DRX-Compass	DSI-DRP
RS	0.68	0.60	0.61	0.63	0.48	0.54
RP	0.56	0.69	0.56	0.53	0.29	0.61
RH	0.58	0.61	0.46	0.52	0.21	0.62
LS	0.57	0.60	0.52	0.61	0.50	0.59
LP	0.65	0.59	0.54	0.56	0.26	0.54
LH	0.54	0.46	0.53	0.53	0.28	0.60
C	0.64	0.74	0.53	0.77	0.48	0.71
Mean	0.60	**0.61**	0.54	0.59	0.36	0.60
SD	0.05	0.08	**0.04**	0.08	0.11	0.05

Values in bold represent the best performance under each metric.

**Table 4:** Distance Measurement Errors (mm) for Different Radiographic Systems.

Marked Pairs		CT	Error of Full-Length Radiographic Systems					
			Eagle Eye	EOS Imaging	UDR780IPRO3F	GC85A	DRX-Compass	DSI-DRP
Plane1	RS-LP	536.14	0.77	3.80	−19.65	−22.46	−38.79	−11.87
	LS-RP	542.83	−8.81	−7.76	−23.63	−24.54	−27.10	−9.65
	RS-RP	463.26	−10.42	−8.56	−22.00	−24.06	−19.89	−4.48
	LS-RS	275.69	−11.03	−15.02	−15.58	−16.94	−30.94	−4.24
	LS-LP	463.30	4.29	7.49	−18.66	−18.54	−34.79	−13.61
	LP-RP	277.23	6.08	9.92	−0.54	−3.17	−4.84	−8.86
Plane2	C-LH	543.17	−12.43	−9.63	−40.67	−33.73	−2.69	−35.93
	C-RH	602.95	−2.70	−2.41	−38.59	−32.18	−3.69	−36.86
	LH-RH	172.42	15.97	19.08	−1.88	−2.39	10.37	−12.42
MAE			**8.05**	9.30	20.13	19.78	19.23	15.32
RMSE			**9.31**	10.49	23.96	22.37	23.46	19.26
Bias			−2.03	**−0.34**	−20.13	−19.78	−16.93	−15.32
SD			**9.09**	10.48	12.99	10.46	16.24	11.67

Values in bold represent the best performance under each metric.

**Table 5:** Statistical Test Results for Measurement Errors.

Radiographic System	Normality *p*	Distribution	Wilcoxon *p*	*t*-Test *p*
Eagle Eye	0.358	Normal	0.570	0.545
EOS Imaging	0.712	Normal	0.910	0.929
UDR780IPRO3F	0.420	Normal	0.004	0.002
GC85A	0.298	Normal	0.004	0.001
DRX-Compass	0.434	Normal	0.027	0.019
DSI-DRP	0.008	Non-normal	0.004	

**Table 6:** Error Source of Three Radiographic Methods.

	Full-Length Radiography	Rotational Stitching	One-Shot	Slot-Scanning
Error Source				
Stitching Errors		Presence of parallax, upper and lower edge distortion	No stitching errors	Distortion and stitching errors decrease as slot width narrows.
Geometric Magnification Errors		Magnification differences lead to inconsistent proportions	Uneven magnification, significant edge geometric distortion	No significant magnification errors
Shooting Angle Errors		Angle deviations between multiple exposures	Non-perpendicular X-ray beam or object tilt causes deformation	No significant angle errors
Motion Artifacts		Patient movement between exposures	Minimal motion artifacts due to single exposure	Line-by-line scanning is easily affected by patient movement
Scattered Radiation		Accumulated scattered radiation from multiple exposures	Large coverage area, significant scattered radiation	Radiation concentrated in the slot area, less scattered radiation
Image Quality		Blurring at stitching areas	Detector resolution may be lower, reducing detail clarity	Affected by slot width and edge quality
System Calibration Errors		Inconsistent parameters across multiple exposures	Detector uniformity and calibration errors	Synchronization issues between slot and detector

## Data Availability

The original contributions presented in this study are included in the article. Further inquiries can be directed to the corresponding authors.

## Abbreviations

The following abbreviations are used in this manuscript:

SID	Source to Image Distance
SOD	Source to Object Distance
HTO	High Tibial Osteotomy
